# Predicting risk of sepsis, comparison between machine learning methods: a case study of a Virginia hospital

**DOI:** 10.1186/s40001-022-00843-4

**Published:** 2022-10-28

**Authors:** Behrad Barghi, Nasibeh Azadeh-Fard

**Affiliations:** Department of Industrial and Systems Engineering, Rochester Institute of Technology, Rochester, NY USA

**Keywords:** Sepsis prediction, Machine learning, Accuracy, Patient data

## Abstract

Sepsis is an inflammation caused by the body's systemic response to an infection. The infection could be a result of many diseases, such as pneumonia, urinary tract infection, and other illnesses. Some of its symptoms are fever, tachycardia, tachypnea, etc. Unfortunately, sepsis remains a critical problem at the hospitals and leads to many issues, such as increasing mortality rate, health care costs, and health care utilization. Early detection of sepsis in patients can help respond quickly, take preventive actions, and prevent major issues. The main aim of this study is to predict the risk of sepsis by utilizing the patient’s demographic and clinical information, i.e., patient’s gender, age, severity level, mortality risk, admit type along with hospital length of stay. Six machine learning approaches, Logistic Regression (LR), Naïve Bayes, Support Vector Machine (SVM), Boosted Tree, Classification and Regression Tree (CART), and Bootstrap Forest are used to predict the risk of sepsis. The results showed that different machine learning methods have other performances in terms of various measures. For instance, the Bootstrap Forest machine learning method exhibited the highest performance in AUC and R-square or SVM and Boosted Tree showed the highest performance in terms of misclassification rate. The Bootstrap Forest can be considered the best machine learning method in predicting sepsis regarding applied features in this research, mainly because it showed superior performance and efficiency in two performance measures: AUC and R-square.

## Highlights

- Six machine learning methods, Logistic Regression (LR), Naïve Bayes, Support Vector Machine (SVM), Boosted Tree, Classification and Regression Tree (CART), and Bootstrap Forest were compared together in order to predict sepsis.
- Early stage of admission data including patient’s gender, age, severity level, mortality risk, admit type along with hospital length of stay were used for predicting sepsis.
- The Bootstrap Forest can be considered the best machine learning method in predicting sepsis regarding applied features in this research mainly because it showed superior performance and efficiency in two performance measures, i.e. AUC and R-square.

## Introduction

Sepsis is a serious medical condition caused by many different organisms, such as bacteria, viruses, and fungi [1]. It occurs when the patient’s body has an abnormal response to an infection. It is a life-threatening situation when the body’s response to infection causes injury to its tissues and organs [2]. It happens when an infection triggers a chain reaction in the whole body. Common signs and symptoms of sepsis are fever, increased heart rate, changes in mental status, and increased respiratory rate. Some factors, such as age (being very young or old), cancer, and diabetes, may increase the risk of sepsis [3]. Septic shock is one of the most serious problems, which occurs when the blood pressure decreases severely due to sepsis. In some high-risk cases, septic shock may not improve after the treatment course and fluid replacement to the body [4]. According to Jawad et al. [5], the mortality rate of sepsis is 30% in normal sepsis, 50% in high severity sepsis, and 80% in septic shock. In the United States and more developed countries, nearly, 30% of people are affected by sepsis each year. Sepsis needs immediate treatment and response to avoid significant concerns and problems. These treatment and response processes are like a complex chain of events, including inflammatory processes, cellular reactions, and circulatory abnormalities [6]. More importantly, a better explanation of diseases and their process will allow for more efficient therapies [7].

Early diagnosis and treatment of sepsis have significant merits like a positive response to antibiotic therapy, a decrease in treatment cost and patient’s length of stay. In this regard, many researchers have made many efforts to identify sepsis in the early stages. Machine learning methods for predicting with learning from supervised or unsupervised data input have been widely used for predicting sepsis [8]. The machine learning models such as Neural Network and Random Forest showed higher performance in predicting the risk of sepsis among emergency patients than traditional screening tools [26].

Logistic Regression is a binary classification model that utilizes a logistic function to forecast binary variables. Logistic Regression is the best choice when the primary goal of developing a model is to assess possible relationships among variables [27]. Some machine learning methods such as SVM and Random Forest have been performed in many fields. However, they may not have the same accuracy in analyzing patient and clinical data [9]. The CART approach has been tried to assess time-varying data. The CART method is powerful for finding predictors without assumptions between variables. In comparison with Logistic Regression analysis, CART, by prioritizing predictive factors, could reveal the interaction among them [10, 11]. Developing predictive models such as machine learning approaches using only the patient’s initial information, like features used in this research, is potentially advantageous, since it can facilitate the physician’s decision and management of patients who may face the risk of sepsis [26]. For example, Li et al. [31], compared 5 machine learning models, SVM, Logistic Regression, Random Forest, K-Nearest Neighbor (KNN), and Gradient Boosting Decision Tree (GBDT), to predict the in-hospital mortality rate in intensive care unit (ICU) patients with sepsis.

### Literature review

The main concentration of this section is to present a literature review of mentioned machine learning methods and their performance in predicting sepsis.

Machine learning methods as powerful tools have been widely used in accurate prediction of sepsis. Fisal et al., developed a Logistic Regression model to predict sepsis utilizing vital signs of patients and blood test results that are available in an early stage of admission [12]. In addition, in the study performed by Mahmud et al. [28] Logistic Regression using Pearson correlation to identify valuable features in predicting sepsis was utilized for early detection of sepsis in ICU patients. In another work done by Wang et al. [29], three machine learning methods, Logistic Regression, SVM, and Logistic model Trees, were applied to predict the onset of sepsis in ICU patients using vital signs and blood culture results. The Logistic model Trees produced better classification performance in comparison with other methods. In another work, Aşuroğlu et al. [13] used Sequential Organ Failure Assessment (SOFA) score to determine the severity of sepsis. They proposed a regression-based analysis by applying seven vital signs obtained in the ICU. They combined Convolutional Neural Network (CNN) features with a Random Forest algorithm for predicting the SOFA score. Deyuan Zhi et al. [14] created a model for predicting in-hospital mortality of sepsis using the MIMIC-III open-source clinical data set. They modified Random Forest and Logistic Regression methods to develop a prediction model of SOFA scores. The Random Forest model with a tenfold cross-validation method was applied by Pirneskoski et al. [15] to show that this machine learning method outperforms the national early warning score for predicting one day mortality. Rodríguez et al. [16] applied four classification methods, SVM, Random Forest, Classification Tree, and Artificial Neural Network (ANN) to predict mortality in adult patients with sepsis. The authors compared these four methods and concluded that SVM and ANN performed better than the other two methods.

In the study which was done by Adhiya [17], different features, such as type of service, place of service, etc., were used to predict the readmission rate using the classification method. In addition, a confusion matrix was applied to obtain the model's accuracy. The classification of gut microbiota of patients in ICU during the sepsis and sepsis shock was done by Liu et al. [18] using binary classifier. The authors concluded that these methods are effective and precise in predicting and monitoring gut microbiota. Mollora et al. [19] utilized recorded electrocardiogram and arterial blood pressure waveforms in the bagged tree classification method to predict sepsis. The results showed that these two factors help predict sepsis in the early stay hours. Optimized Random Forest applied by Lyra et al. [20] to predict sepsis for imbalanced data from ICU.

A Bagged Decision Tree as a machine learning method with highly unbalanced misclassification cost was provided by Firoozabadi et al. [21], which had 15 features for forecasting sepsis. Doggart et al. [22] applied random under sample (RUS) Boosted Tree to classify sepsis from ICU data. The model showed sensitivity and specificity of 53.4% and 83.6%, respectively. Stepwise multiple Logistic Regression (MLR) analysis and CART analysis were performed by Metsvaht et al. [23] to analyze data of maternal and early neonatal characteristics predicting failure of empiric antibiotic treatment which derived from univariate Logistic Regression analysis. In another study by García-Gallo et al. [24] utilized a machine learning model based on stochastic gradient boosting for predicting 1 year mortality in patients with sepsis. The receiver operating characteristic curve was drawn for the evaluation of the model. A discrete conditional survival model (DC-S) was introduced in the work of Marshall [25] with the Classification Tree, Logistic Regression, and Naïve Bayes classification components to predict the length of stay of the babies and sepsis using the baby characteristics known on the first day of admission. KNN, Random Forest, Logistic Regression, Decision Tree, Gradient Boosting, and Naïve Bayes machine learning methods were used by Metskera et al. [30] to predict sepsis in patients hospitalized in the ICU for at least one month. The results showed that the severity of sympathicotonia, XII blood coagulation factor, total protein increase in LH, prolactin, increased natriuretic peptide, a decrease of albumin, cortisol, an increase of fibrinogen, the index of the APTT are the main factors that affect the risk of sepsis. Again Li et al. [31] applied five machine learning methods to predict in-hospital mortality in patients with sepsis. GBDT method showed superior performance with the highest area under the ROC curve (0.992).

As presented above in the work of Doggart et al. [22], Metskera et al. [30], and Li et al. [31] many types of research have been performed to predict mortality risk in a patient with sepsis in critical places like the ICU by utilizing different features, such as blood factors, heart rate, cortisol, APTT, etc. Besides, Fisal et al. [12], showed that several research have been done to forecast risk of sepsis with data from early stages of admission-like blood pressure. Machine learning models, such as SVM, Naïve Bayes, and Random Forest, as novel approaches to predict the risk of sepsis with significant performance, have been widely used in those researches. Early diagnosis and treatment of sepsis have considerable merits, such as a positive response to antibiotic therapy, decreasing treatment cost, and patient’s length of stay. In addition, Kijpaisalratana et al. [26] concluded that predicting the risk of sepsis using only the patient’s initial information, like features used in this research, is potentially advantageous because it can facilitate the physician’s decision and management of patients who may face the risk of sepsis. Therefore, in this paper, by utilizing six machine learning approaches, Logistic Regression, Naïve Bayes, SVM, Boosted Tree, CART, and Bootstrap Forest and by considering the early stage of admission data: patient’s gender, age, severity level, mortality risk, admit type along with hospital length of stay aimed to predict the risk of sepsis which is one of the most important and critical issues that many patients face during their treatment course. One of the most significant contributions of the current study is that the data set used for anticipating sepsis is imbalanced. The imbalanced data can lead to bias in results. By considering this fact, this research aims to examine the performance of various machine learning methods in anticipating sepsis.

The rest of the paper is structured as follows: the methodology used in this paper is presented in the Methodology section. In the Results section, the results of the experiments and a discussion about the results are provided. Finally, in Conclusions section the concluding points of this paper are presented.

## Methodology

In this section, the methodology that has been utilized to address the problem statement while achieving the objectives of the study is presented. The structure of this section is organized as follows. At first, the data extraction process for the survey is provided, then the analysis techniques utilized to predict sepsis are presented.

According to the Centers for Disease Control and Prevention (CDC), at least 1.7 million adults in the US develop sepsis, and among them, about 270,000 people die because of sepsis. The rate of septicemia death in Virginia state is 10.6 per 1000 people in 2019. Therefore, it becomes essential to reduce the septic rate as much as possible to prevent the danger of death in septic patients. Hence, the data collected from a large teaching hospital in Virginia state with 506 beds were utilized and considered a case study for performing experiments in forecasting sepsis. This data set consists of initial information about sepsis and non-sepsis patients in the mentioned hospital between Oct 2012 and Sep 2014. As stated in the past sections, early sepsis prediction can prevent irreversible consequences, such as death. Many researchers have provided machine learning methods to overcome this problem. SVM, CART, Naïve Bayes method, Bootstrap Tree, and ANN are the well-known machine learning methods that researchers have widely used. Some specific features, hyper-parameters, and data sets were applied to explain and forecast sepsis's risk in the mentioned methods.

In this study, six well-known machine learning algorithms, Logistic Regression, Naïve Bayes, SVM, Boosted Tree, CART. Bootstrap Forest is developed to classify and predict the risk of sepsis by considering six patient initial information features: patient’s gender, age, hospital length of stay, severity level, mortality risk, and admit type. The K-fold cross validation method with *K* = 10 means a 90% training data set and a 10% validation data set are applied to examine the accuracy and increase the probability of success in the models. To prevent overfitting in Trees, the pruning method is utilized. In addition, confusion matrix, ROC, and Lift curves for models were obtained to show the final accuracy and validation of proposed models. Besides, other factors such as accuracy, specificity, precision, recall, F-1 score, the area under the ROC curve (AUC), misclassification rate, and R-square were also calculated to show the performance of the models. Regarding the applied parameters, models were compared to each other to see which model had better results among all proposed models to predict and explain the risk of sepsis.

Ten SVMs with a cost between 0.01 and 5 and gamma between 0.001 and 0.5 were run, and the best model was chosen to show here. The specification of the best model is Cost: 4.64 and Gamma: 0.363. The Bootstrap Forest consists of 100 trees, minimum split per tree of 10 and maximum split of 2000, and a minimum size split of 21. The Boosted Tree also includes 17 splits per tree, a learning rate of 0.1, overfit penalty of 0.0001, a minimum size split of 5, and several layers of 200.

Once the data were cleaned, it was found that a total of 20,005 records of the patients were available. It should be pointed that the people who died during the hospitalization were omitted from the data set and the data set did not include missing data. About 1486 (7.48%) of patients were infected with sepsis over the mentioned period. The descriptive statistic of the patients regarding proposed features is calculated in Table 1. In addition, the sepsis rate of each feature is presented in this table.

**Table 1:** Descriptive statistics

Features	level	Sepsis rate
Gender	female	4.82
	male	2.66
Severity level	minor	0.10
	moderate	1.17
	major	2.84
	extreme	3.37
Mortality risk	minor	0.59
	moderate	1.38
	major	2.44
	extreme	3.07
Admit type	emergency	6.07
	urgent	1.20
	elective	0.20
Length of stay	1–5 days	5.72
	6–10 days	1.14
	11–20 days	0.52
	21–30 days	0.06
	31–40 days	0.03
	40 +	0.00
Age	1–10 years	0.00
	11–20 years	0.00
	21–30 years	0.00
	31–40 years	0.32
	41–50 years	0.00
	51–60 years	0.44
	61–70 years	0.7
	71–80 years	1.42
	80 + years	4.59

## Results

As shown in Table 1, most people who suffered from sepsis had both severity level and mortality risk of “extreme.” This result could be predictable, because when people have the situation “extreme” in mortality risk and severity levels are more prone to sepsis. In addition, their admit type is emergency. This implies that most people prone to sepsis are admitted in an emergency condition. The length of stay shows that most people who suffer from sepsis have a length of stay between 1 and 5 days. Females are more likely to have sepsis than males. According to the data, people over 70 years are more prone to get sepsis, which was predictable.

The six machine learning methods with tenfold cross-validation are shown in Table 2. It included the AUC, precision, recall, accuracy, F1 score, R-square, and misclassification rate. The AUC ranged from 0.899 to 0.937 for the six predictive models. Bootstrap Forest shows the largest AUC (0.908). Two other classification methods, based on trees, also show an AUC greater than 0.9. Besides, SVM with 0.829 shows the smallest AUC among all methods. This implies that machine learning methods based on trees offer better performance than other methods in terms of AUC. Accuracy is mainly used when the classes are balanced (that is, each label has about the same number of occurrences), and there is no significant downside to predicting false negatives. However, it is misleading for imbalanced classes. Therefore, due to the imbalanced data set used in this research, this performance measure cannot provide reliable and accurate results.

**Table 2:** Results of six proposed machine learning methods

	Logistic Regression	Boosted Tree	Bootstrap Forest	CART	SVM	Naïve Bayes
Accuracy	0.933	0.936	0.935	0.933	0.937	0.899
Specificity	0.984	0.983	0.984	0.986	0.983	0.923
precision	0.607	0.634	0.620	0.639	0.641	0.385
Recall	0.297	0.349	0.323	0.282	0.362	0.385
F-1 score	0.399	0.450	0.425	0.391	0.463	0.468
Misclassification rate	0.066	0.063	0.064	0.066	0.063	0.100
AUC for training data	0.887	0.901	0.908	0.901	0.829	0.884
R-square	0.327	0.355	0.366	0.354	0.223	0.033

Contrary to AUC, SVM shows the maximum accuracy among all methods (0.937). However, this difference is negligible. Therefore, it can be concluded that all methods offer the same accuracy except the Naïve Bayes method, which has the lowest accuracy (0.899). The F1 score is also used when the classes are imbalanced, and there is a severe downside to predicting false negatives. Because this study uses an imbalanced data set, the result of this performance measure can be more accurate and reliable. The Naïve Bayes method has the most significant F-1 score among all methods with little difference from SVM, which stand in second place. Both CART and Logistic Regression show the smallest F-1 score. Besides, the results exhibit that the F-1 scores for all the models are lower than the ideal score, and the probable reason for low F-1 scores could be the type of data set being analyzed. As the data are actual data from the healthcare industry, F-1 scores could be low. R-squared is a statistical measure representing the proportion of the variance for a dependent variable explained by an independent variable or variables in a model. Therefore, in terms of R-square, the bootstrap can explain 0.366 of the variances of the sepsis, and Naïve Bayes can explain only 0.033 of the variances of the sepsis. Lift is the proportion of the predicted rate to the average rate. The method with the lift curve with the highest maximum lift point (first point on the right side of the curve) shows better performance. By looking at the lift curves of the proposed methods, it can be concluded that all models represent the same performance. Different plots and curve such as ROC, lift curves, and R-square history plots of all proposed methods along with comparison graph which can help to find the best model with minimum misclassification rate by considering two hyperparameters gamma and cost in the SVM method, are presented in the appendix.

## Discussion

The present study evaluated different machine learning methods which have been currently found that can be considered powerful tools in predicting and explaining the risk of sepsis using data from various places and stages at the hospital. Early hospital stages data of patients can give a wide range of information to the physicians and doctors for anticipating the risk of sepsis that patients may face. Therefore, this initial information was used as an input for the machine learning methods to predict and explain sepsis.

The initial data analysis showed that females are about twice as likely as males to have sepsis during their hospitalization period. This result is inconsistent with the outcome of Pietropaoli et al. [32] that sepsis is higher in males than females. This is mainly due to features used in that research and the place, where sepsis was measured and diagnosed in the hospital.

Severity level, mortality risk, and admit type are three features that show the intensity of the patient's condition in the admission process. With the analysis of these features, it can be concluded that, in general, patients who are admitted to extreme conditions are more likely to infect with sepsis than other patients. This result was predictable, because these patients have more severe medical problems; as a result, they need more accurate and intense care. Meanwhile, their body shows less strength to different diseases and infections such as sepsis due to less physical strength. In addition, altering one situation in these patients leads to severe damages, such as sepsis. In another work, Angele et al. [33] showed that the female gender demonstrates more protection for septic conditions. In contrast, the male gender may show less strength due to a decreased cell-mediated immune response and cardiovascular functions. Male sex hormones, i.e., androgens, are more suppressive on cell-mediated immune responses.

But on the contrary, female sex hormones demonstrate protective impacts, which might contribute to the natural merits of females under septic conditions. Patients aged more than 80 years had more sepsis. From a medical point of view, this result can be interpreted, so that patients at this age have less physical strength and are more prone to various diseases and infections. In terms of length of stay, due to the fact that the majority of patients were hospitalized for the range between 1 and 10 days, more sepsis was observed for patients with a length of stay in this range. More investigation should be done to examine the effect of patient length of stay on the risk of sepsis.

**Fig. 1:**
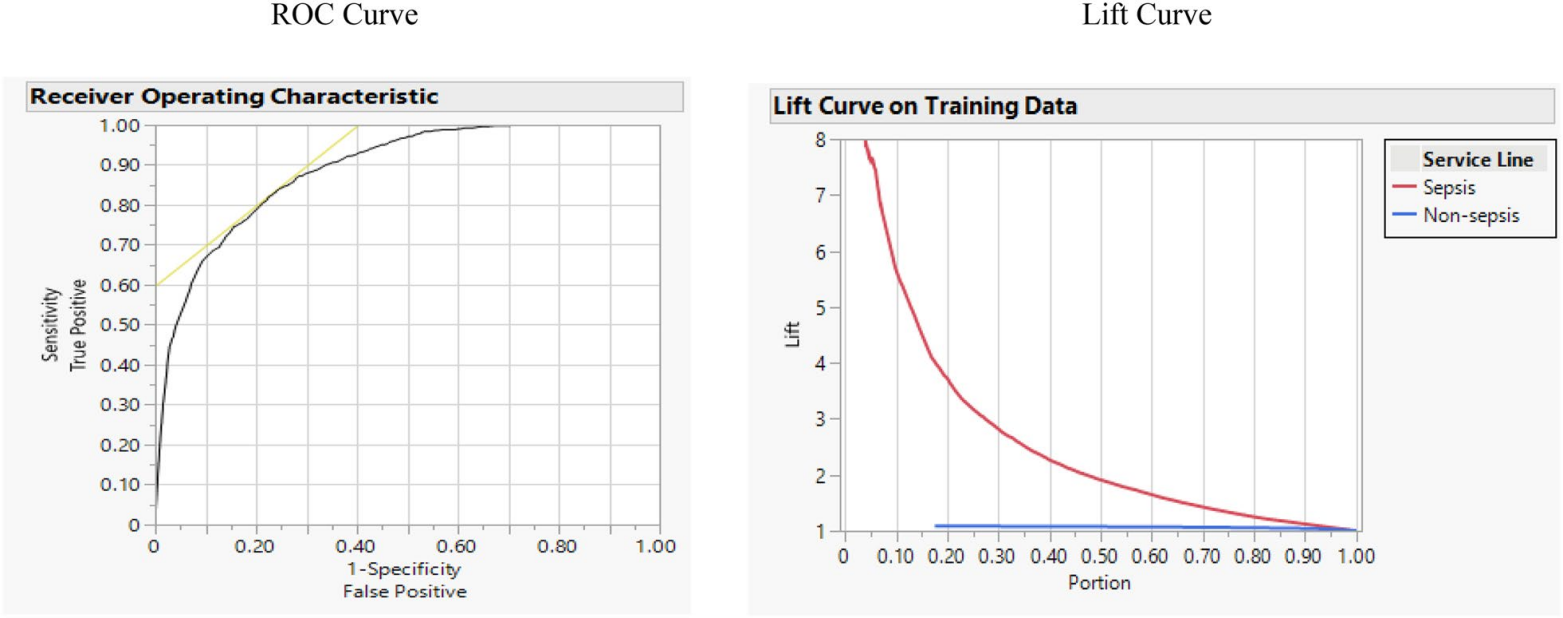
ROC and lift curve for Logistic Regression

Logistic Regression is a statistical method for predicting binary outcomes, and it is also a supervised machine learning algorithm developed for classification problems. This algorithm has been widely used to predict the risk of sepsis by utilizing various data sets, initial patient information [12], ICU information [28], among others. Logistic Regression is more effective in predicting mortality, according to Cheng et al. [34]. Although other machine learning methods exhibited higher performance and more efficient results, the results of Logistic Regression for the current study were acceptable enough (accuracy: 0.933, F-1 score: 0.399, misclassification rate: 0.066, AUC: 0.887, and R-square: 0.327). Figure 1 shows the ROC and Lift curve of the Logistic Regression method.

**Fig. 2:**
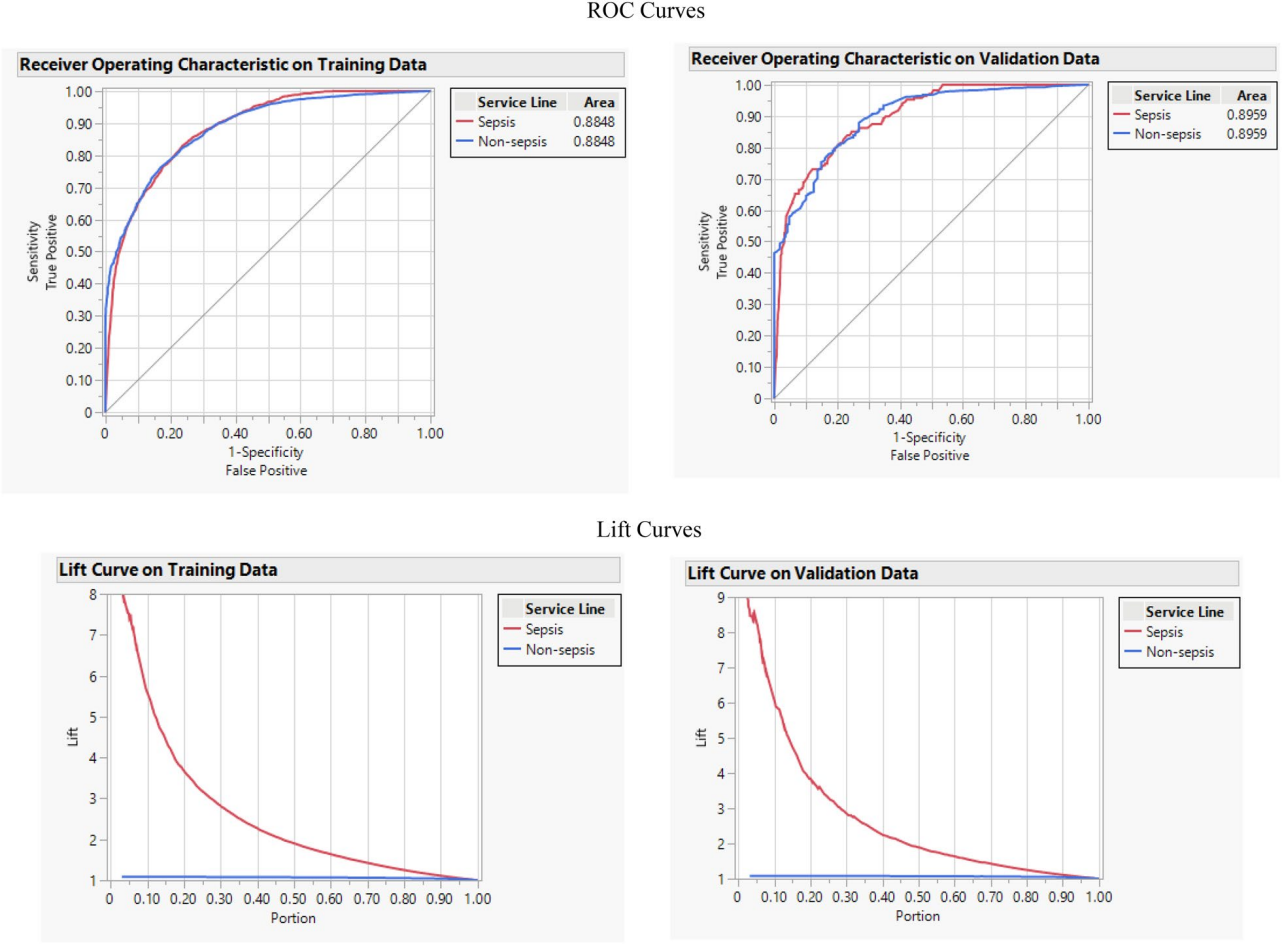
ROC and lift curve Naïve Bayes

Naïve Bayes is a supervised classification algorithm based on the Bayes Theorem, which assumes that all predictor variables are independent. As a result, it tends to be a highly sophisticated probability-based machine learning algorithm. Large data sets can be easily analyzed using it as well. Since the data set used in this research is imbalanced, the F-1 score as a performance measure that shows high efficiency and accuracy in the imbalanced data set can be a reliable factor for evaluating the model. The Naïve Bayes method exhibited the highest F-1 score among all algorithms (0.468). With a negligible difference to Naïve Bayes, SVM by F-1 score of 0.463 lay down in the second place. However, this algorithm had the highest misclassification rate among all algorithms (0.100). This algorithm's other performance measures are accuracy: 0.899, AUC: 0.884, R-square: 0.033. In addition, Fig. 2 shows the ROC and Lift curve of the Naïve Bayes algorithm.

**Fig. 3:**
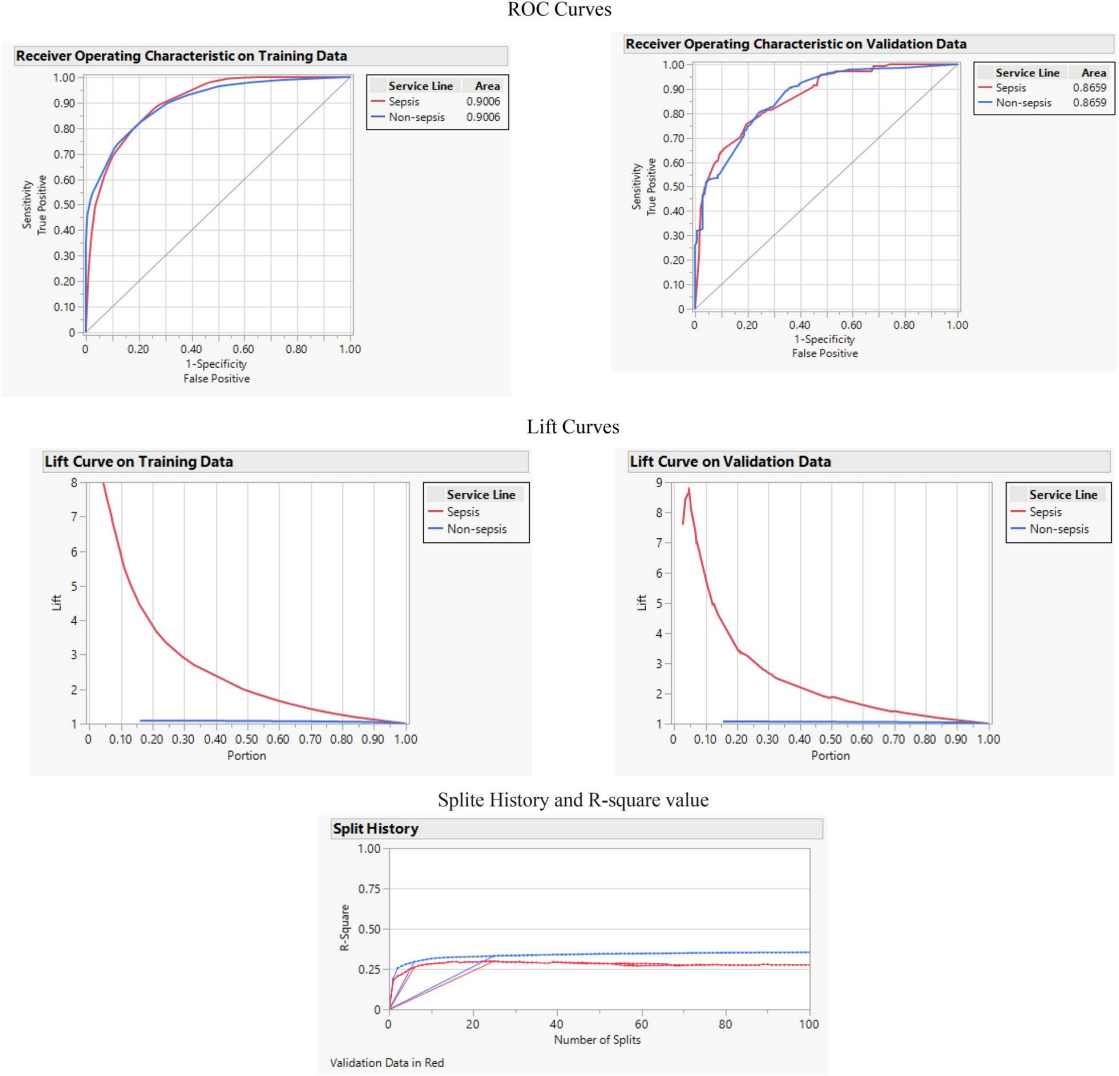
ROC, lift, and cumulative curves for CART

CART algorithm utilizes a decision tree to go from observations about a feature (represented in the branches) to conclusions about the target value (represented in the leaves). This algorithm is widely used for non-linear data sets, which have various features in such a way that partitioning the data space and fitting a simple prediction model within each partition. A single CART that has too good a result may overfit the data. To prevent overfitting, the pruning method is used to limit the depth of the tree. This method has been widely used to classify and predict binary outcomes, such as mortality risk of sepsis in the ICU [24]. Performance measures related to CART algorithm are as follows: accuracy: 0.933, F-1 score: 0.391, misclassification rate: 0.066, AUC: 0.901, and R-square: 0.354. Figure 3 shows the ROC and Lift curve and the split history curve that shows R-square variations in each split.

**Fig. 4:**
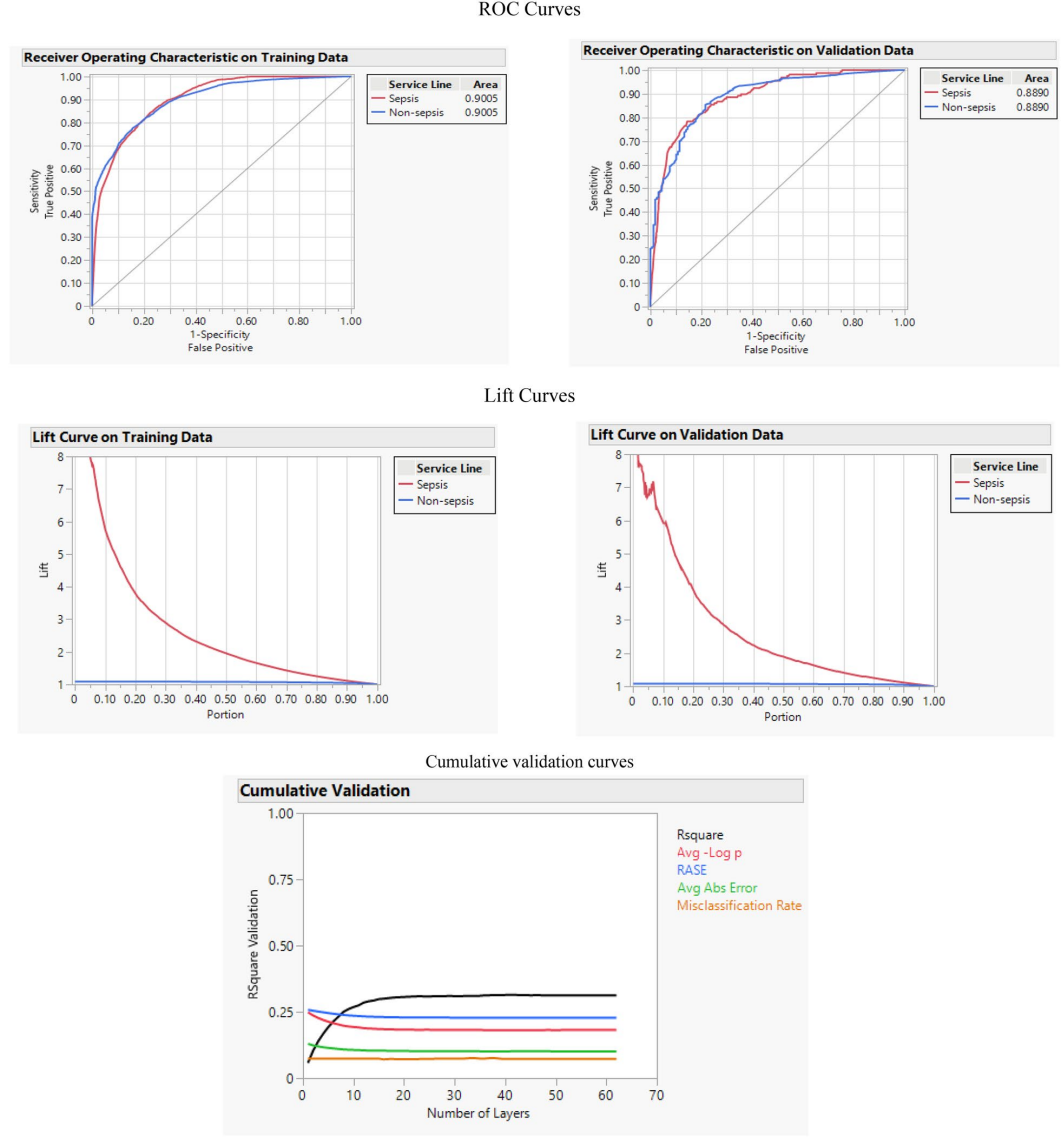
ROC, lift, and cumulative curves for Boosted Tree

Boosted Tree algorithm also uses decision trees and the boosting method to improve the model’s performance and prevent overfitting using multiple weak learners. This algorithm uses decision trees with slightly higher performances than chance. As part of the boosting method, a data set is explicitly treated as a numerical optimization problem with gradient descent. In the current study, the Boosted Tree algorithm and SVM showed the least misclassification rate among all algorithms (0.063). Other performance measures of this algorithms are as follows: accuracy: 0.936, F-1 score: 0.450, AUC: 0.901, and R-square: 0.355. Figure 4 shows the ROC and Lift curve of this algorithm and the cumulative validation curve, which exhibits various performance factors such as R-square or random average square error (RASE) in each layer of the tree.

**Fig. 5:**
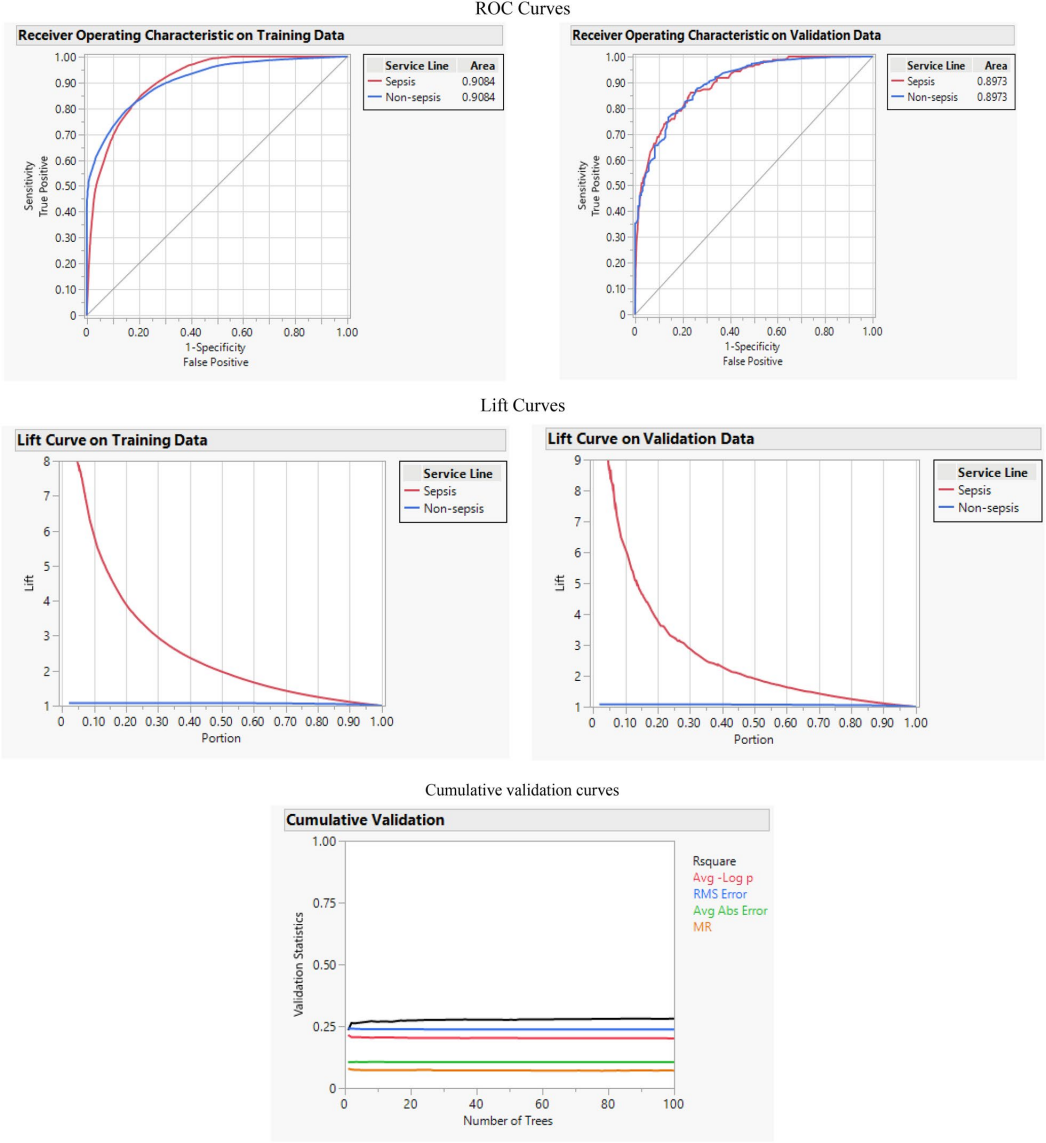
ROC, lift, and cumulative curves

Ensemble machine learning is a machine learning that involves training multiple weak learners on the same problem and then combining those results for better accuracy and efficiency. The Bootstrap Forest is a machine learning algorithm that increases the accuracy of a model using ensemble learning. In this method, the sample of the training data set is created utilizing the bootstrap method, which involves choosing examples randomly with replacements. Some original examples may not be used, and some may be used more than once. In the end, the final decision is made by averaging the N learners (here trees). In this study, Bootstrap Forest shows the highest AUC and R-square among all algorithms by 0.908 and 0.366, respectively. This algorithm's accuracy, F-1 score, and misclassification rate are 0.935, 0.425, and 0.064. Figure 5 shows the ROC and Lift curve of this algorithm and the cumulative validation curve, which exhibits various performance factors, such as R-square or RASE in each tree.

**Fig. 6:**
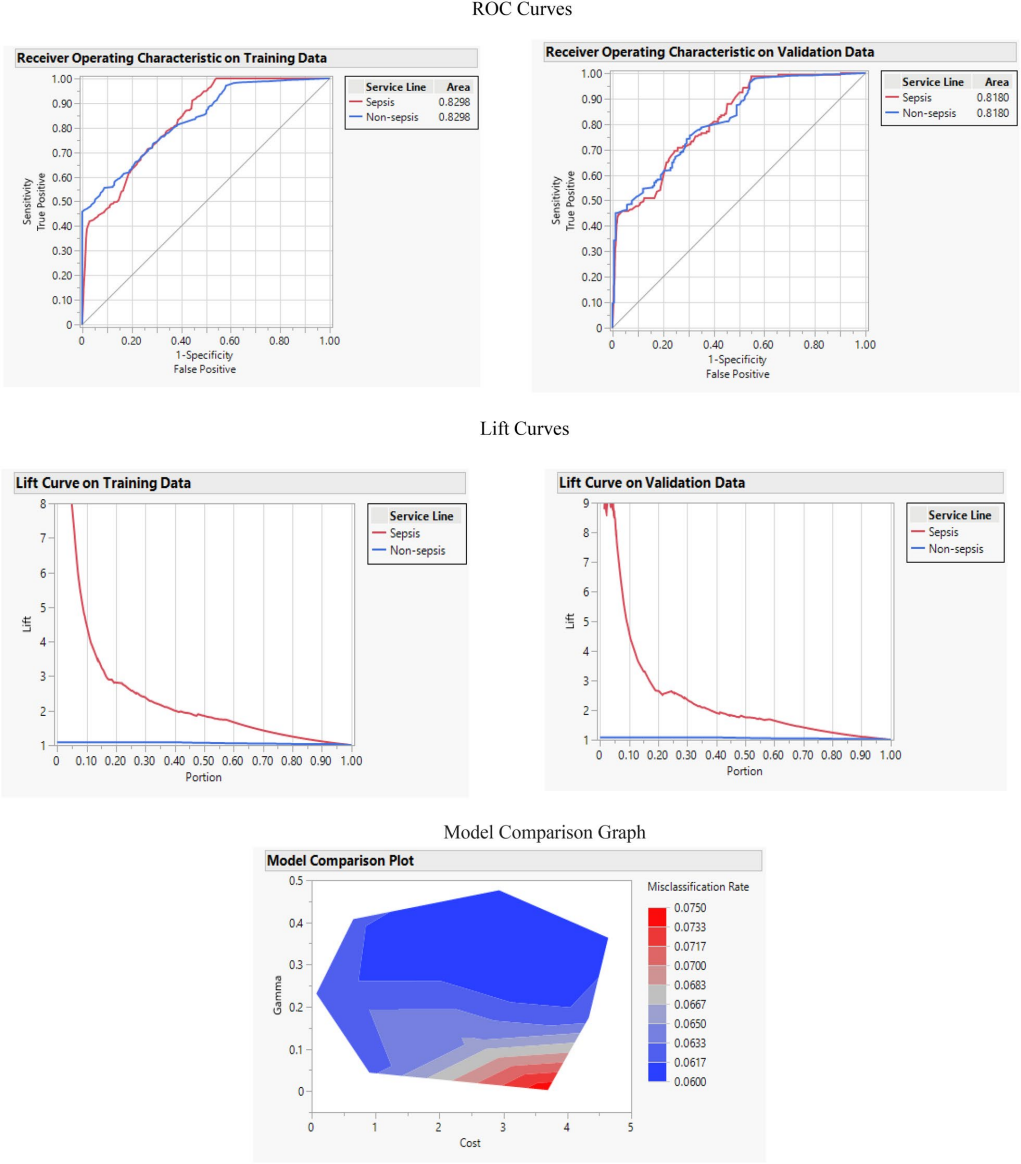
ROC, lift, and model comparison graph for SVM

SVM algorithm is a supervised machine learning algorithm that creates the best line or decision boundary to separate n-dimensional space into classes. The new data point can be put in the correct class. This best decision boundary is called a hyperplane. SVM chooses the extreme points/vectors, called super vectors, to help create the hyperplane. SVM was first proposed by Cortes et al. [35]. They used statistical learning theory to create SVM. The main advantage of SVM is that it uses a kernel function to minimize both model complexity and prediction error. SVM showed the highest accuracy by 0.937 and the least misclassification rate with Boosted Tree algorithm by 0.063. The F-1 score of SVM was 0.463, which was the highest F-1 score after the Naïve Bayes algorithm. The AUC and R-square of the SVM algorithm were 0.829 and 0.223, respectively. Figure 6 shows the ROC and Lift curve of the SVM algorithm. In addition, the model comparison graph is provided to exhibit the best gamma value and cost value that creates the best SVM algorithm with the highest performance and efficiency.

This work has some limitations. First, the study was performed at a single hospital data; the performance of machine learning methods may be different when applied to a sample of various hospitals with more other features. Second, the data are unbalanced. Therefore, some performance factors such as accuracy that reduced efficiency when applied to the unbalanced data might be biased toward 1 class with a higher population. Third, determining the applicability and usefulness of different machine learning methods requires independent and external validation in a sample population that is entirely different from the current sample.

## Conclusions

In this study, predicting the risk of sepsis using early hospital stages data of patients were investigated. The data consist of the patient’s gender, age, severity level, mortality risk, admit type, and hospital length of stay. To serve this purpose, six machine learning methods, Logistic Regression, Naïve Bayes, SVM, Boosted Tree, CART, and Bootstrap Forest were applied. The efficiency of each method was evaluated by five performance measures; accuracy, F-1 score, misclassification rate, R-square, and AUC. One crucial point that should be considered is that because the utilized data are imbalanced and accuracy for imbalanced information is biased toward the group with a higher population, this performance measure cannot be considered as a reliable measure for evaluation of the mentioned methods. However, the F-1 score is mainly used to evaluate methods that used imbalanced data are of interest. Among all machine learning methods, SVM showed the highest accuracy by 0.937 and the lowest misclassification rate and Boosted Tree by 0.063. The Naïve Bayes method exhibited the highest F-1 score at 0.468, followed by SVM with an F-1 score of 0.463. In addition, the Bootstrap Forest exhibited the highest AUC and R-square among all algorithms by 0.908 and 0.366, respectively. In terms of this lift curve, all applied machine learning methods have high performance and efficiency to predict and explain the risk of sepsis regarding mentioned features. The Bootstrap Forest can be considered the best machine learning method in predicting sepsis regarding applied features in this research mainly, because it showed superior performance and efficiency in two performance measures: AUC and R-square.

## Data Availability

The data sets used and/or analyzed during the current study are available from the corresponding author on reasonable request.
